# Supplementary material for: Modelling the impact of clot fragmentation on the microcirculation after thrombectomy
Source: PLoS Comput Biol. 2021 Mar 12;17(3):e1008515. doi: 10.1371/journal.pcbi.1008515 (PMC7990195; doi:10.1371/journal.pcbi.1008515)
Supplement: S1 Text — (PDF) [file pcbi.1008515.s001.pdf]

S1 Appendix – Accounting for Fåhræus-Lindqvist effect

The Fåhræus-Lindqvist effect is accounted for here through a correction for in-vivo viscosity taking into account the endothelial surface layer:

$$\mu_{vitro} = \mu_p \left[1 + (\eta_{0.45} - 1)\frac{(1 - H_{ct})^{C_D} - 1}{(1 - 0.45)^{C_D} - 1}\right] \quad (1)$$

$$\eta_{0.45} = 220e^{-1.3D} + 3.2 - 2.44e^{-0.06D^{0.645}} \quad (2)$$

$$C_D = (0.8 + e^{-0.075D})\left(-1 + \frac{1}{1 + 10^{-11}.D^{12}}\right) + \frac{1}{1 + 10^{-11}.D^{12}} \quad (3)$$

$$\mu_{vivo} = \mu_{vitro}.\left(\frac{D}{D_{eff}}\right)^4 \quad (4)$$

where μ_p is the viscosity of blood plasma (1.2 mPa s), μ_{vitro} is the in-vitro blood viscosity, μ_{vivo} is the apparent in-vivo viscosity, and $\eta_{0.45}$ is the relative apparent viscosity of blood for a discharge haematocrit of 0.45. H_{ct} is the discharge haematocrit, set here to be 0.45. The diameter of the blood vessel is D (μm) and D_{eff} is the effective diameter of the blood vessel that takes into account the thickness of the endothelial surface layer, as derived using correlations detailed in Pries & Secomb [1].

References

[1] A. R. Pries and T. W. Secomb, "Microvascular blood viscosity in vivo and the endothelial surface layer," *Am J Physiol Hear. Circ Physiol*, vol. 289, no. 6, pp. H2657-64, 2005, doi: 10.1152/ajpheart.00297.2005.
