# Supplementary material for: Modelling the impact of clot fragmentation on the microcirculation after thrombectomy
Source: PLoS Comput Biol. 2021 Mar 12;17(3):e1008515. doi: 10.1371/journal.pcbi.1008515 (PMC7990195; doi:10.1371/journal.pcbi.1008515)
Supplement: S2 Text — (PDF) [file pcbi.1008515.s002.pdf]

S2 Appendix – Correlation Matrix Distance

We use the correlation matrix difference (CMD) to compare the covariances of distributions against each other. The CMD is defined as [1]:

$$d_{corr}(\boldsymbol{R_1}, \boldsymbol{R_2}) = 1 - \frac{tr\{\boldsymbol{R_1}\boldsymbol{R_2}\}}{\|\boldsymbol{R_1}\|_f \|\boldsymbol{R_2}\|_f} \quad (1)$$

Where d_{corr} is the distance metric, $\boldsymbol{R_1}$ and $\boldsymbol{R_2}$ are the two covariance matrices, $tr\{\boldsymbol{R_1}\boldsymbol{R_2}\}$ the trace of the product of the two covariance matrices and $\|.\|_f$ is the Frobenius norm. The CMD has a value of 0 when the covariance matrices are identical and a value of 1 when they are orthogonal.

References

1. Herdin M, Czink N, Özcelik H, Bonek E. Correlation matrix distance, a meaningful measure for evaluation of non-stationary MIMO channels. IEEE Vehicular Technology Conference. 2005. pp. 136–140. doi:10.1109/vetecs.2005.1543265
