# Supplementary material for: Modelling the impact of clot fragmentation on the microcirculation after thrombectomy
Source: PLoS Comput Biol. 2021 Mar 12;17(3):e1008515. doi: 10.1371/journal.pcbi.1008515 (PMC7990195; doi:10.1371/journal.pcbi.1008515)
Supplement: S1 Table — The first 4 layers can be approximated as log-normal. The p-value is the Mann-Whitney U-test between adjacent layers to determine if adjacent layers have similar distributions of permeability. For comparison, the capillary bed permeability is 4.28x10^-4^ mm3 s kg-1. (DOCX) [file pcbi.1008515.s003.docx]

**S1 Table. The main statistics for the distribution of permeabilities in each layer of the healthy voxels (for 100 voxels).** The first 4 layers can be approximated as log-normal. The p-value is the Mann-Whitney U-test between adjacent layers to determine if adjacent layers have similar distributions of permeability. For comparison, the capillary bed permeability is 4.28x10^-4^ mm^3^ s kg^-1^

| **Layer** | **Mean (mm^3^ s kg^-1^)** | **Median (mm^3^ s kg^-1^)** | **SD (mm^3^ s kg^-1^)** | **IQR (mm^3^ s kg^-1^)** | **Between adjacent layers p-value (Mann Whitney U test)** |
| --- | --- | --- | --- | --- | --- |
| 1 | 0.0221 | 0.0200 | 0.0107 | 0.0119 | $\boldsymbol{\}}$ < 0.001  $\boldsymbol{\}}$ 0.5  $\boldsymbol{\}}$ < 0.001  $\boldsymbol{\}}$ < 0.0001  $\boldsymbol{\}}$ < 0.0001 |
| 2 | 0.0291 | 0.0231 | 0.0171 | 0.0152 |  |
| 3 | 0.0314 | 0.0248 | 0.0221 | 0.0216 |  |
| 4 | 0.0246 | 0.0189 | 0.0218 | 0.0203 |  |
| 5 | 0.0129 | 0.008 | 0.0132 | 0.0150 |  |
| 6 | 0.0036 | 0.0020 | 0.0044 | 0.0040 |  |
