# Supplementary material for: Modelling the impact of clot fragmentation on the microcirculation after thrombectomy
Source: PLoS Comput Biol. 2021 Mar 12;17(3):e1008515. doi: 10.1371/journal.pcbi.1008515 (PMC7990195; doi:10.1371/journal.pcbi.1008515)
Supplement: S2 Table — The first 4 layers can be approximated as log-normal. The p-value is the Mann-Whitney U-test between adjacent layers to determine if adjacent layers have similar distributions of coupling coefficients. (DOCX) [file pcbi.1008515.s004.docx]

**S2 Table. The main statistics for the distribution of coupling coefficients in each layer of the healthy voxels (for 96 voxels).** The first 4 layers can be approximated as log-normal. The p-value is the Mann-Whitney U-test between adjacent layers to determine if adjacent layers have similar distributions of coupling coefficients.

| **Layer** | **Mean (Pa^-1^ s^-1^) x10^-4^** | **Median (Pa^-1^ s^-1^) x10^-4^** | **SD (Pa^-1^ s^-1^) x10^-4^** | **IQR (Pa^-1^ s^-1^) x10^-4^** | **Between adjacent layers p-value (Mann Whitney U test)** |
| --- | --- | --- | --- | --- | --- |
| 1 | 0.56 | 0.28 | 0.72 | 0.54 | $\boldsymbol{\}}$ < 0.001  $\boldsymbol{\}}$ 0.31  $\boldsymbol{\}}$ < 0.0001  $\boldsymbol{\}}$ < 0.0001  $\boldsymbol{\}}$ < 0.0001 |
| 2 | 0.69 | 0.44 | 0.75 | 0.50 |  |
| 3 | 0.59 | 0.43 | 0.51 | 0.50 |  |
| 4 | 0.39 | 0.27 | 0.35 | 0.36 |  |
| 5 | 0.17 | 0.13 | 0.20 | 0.20 |  |
| 6 | 0.08 | 0.04 | 0.10 | 0.10 |  |
