# Supplementary figures and images for: Modelling the impact of clot fragmentation on the microcirculation after thrombectomy
Source: PLoS Comput Biol. 2021 Mar 12;17(3):e1008515. doi: 10.1371/journal.pcbi.1008515 (PMC7990195; doi:10.1371/journal.pcbi.1008515)

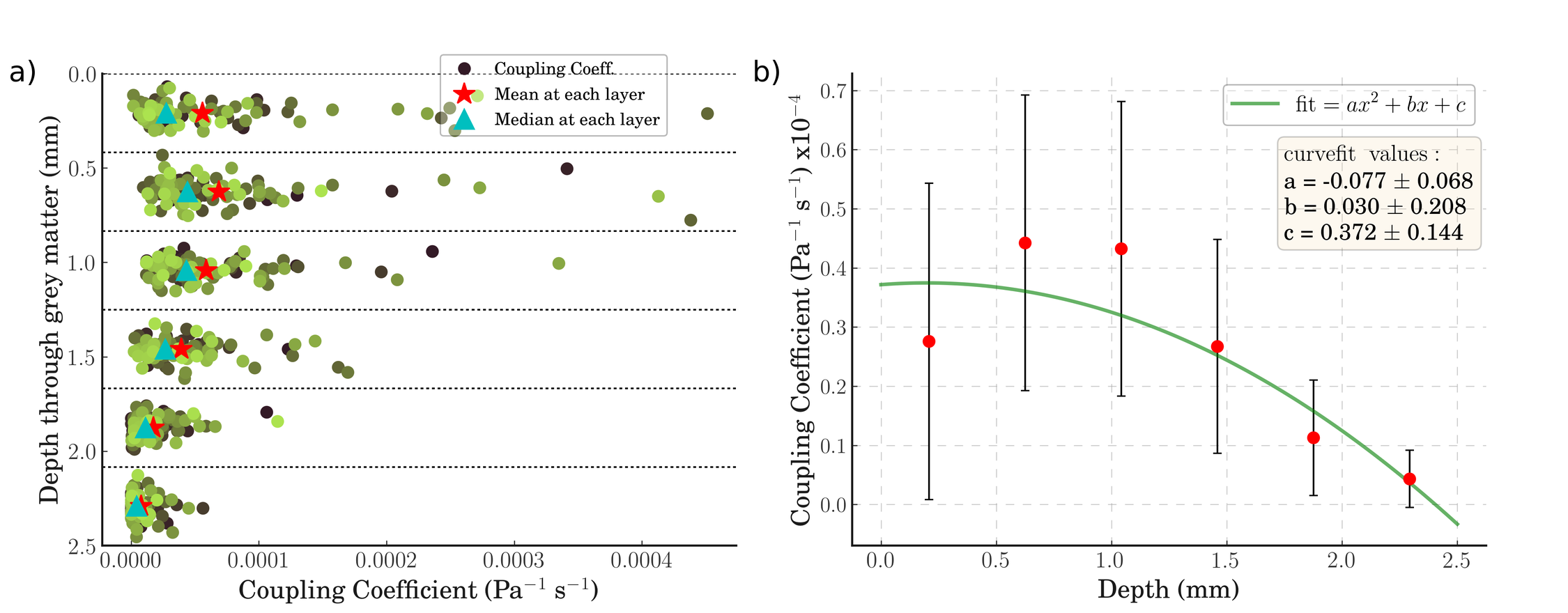

Supplement: S1 Fig — a) A scatter plot of the coupling coefficients of the 100 voxels at each of the 6 depth layers. The mean arteriolar coupling coefficient is indicated with a red star at each layer. b) A quadratic line of best fit over the median coupling coefficients at each layer–error bars are interquartile ranges. (TIF) [file pcbi.1008515.s005.tif]

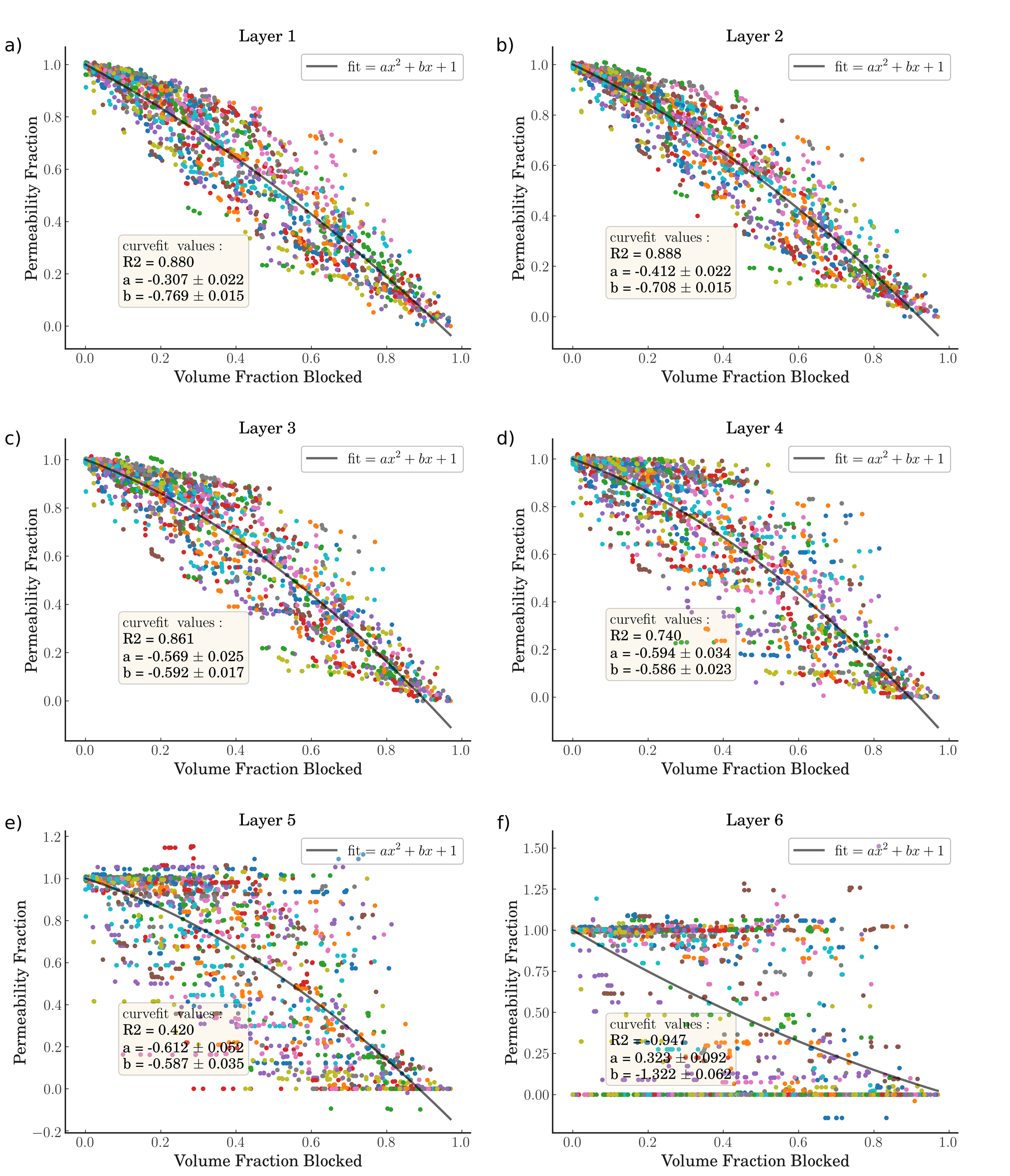

Supplement: S2 Fig — a-f) The drop in permeability with respect to volume fraction blocked over the 6 layers, starting with the top layer a) and ending at the bottom layer f). (TIF) [file pcbi.1008515.s006.tif]

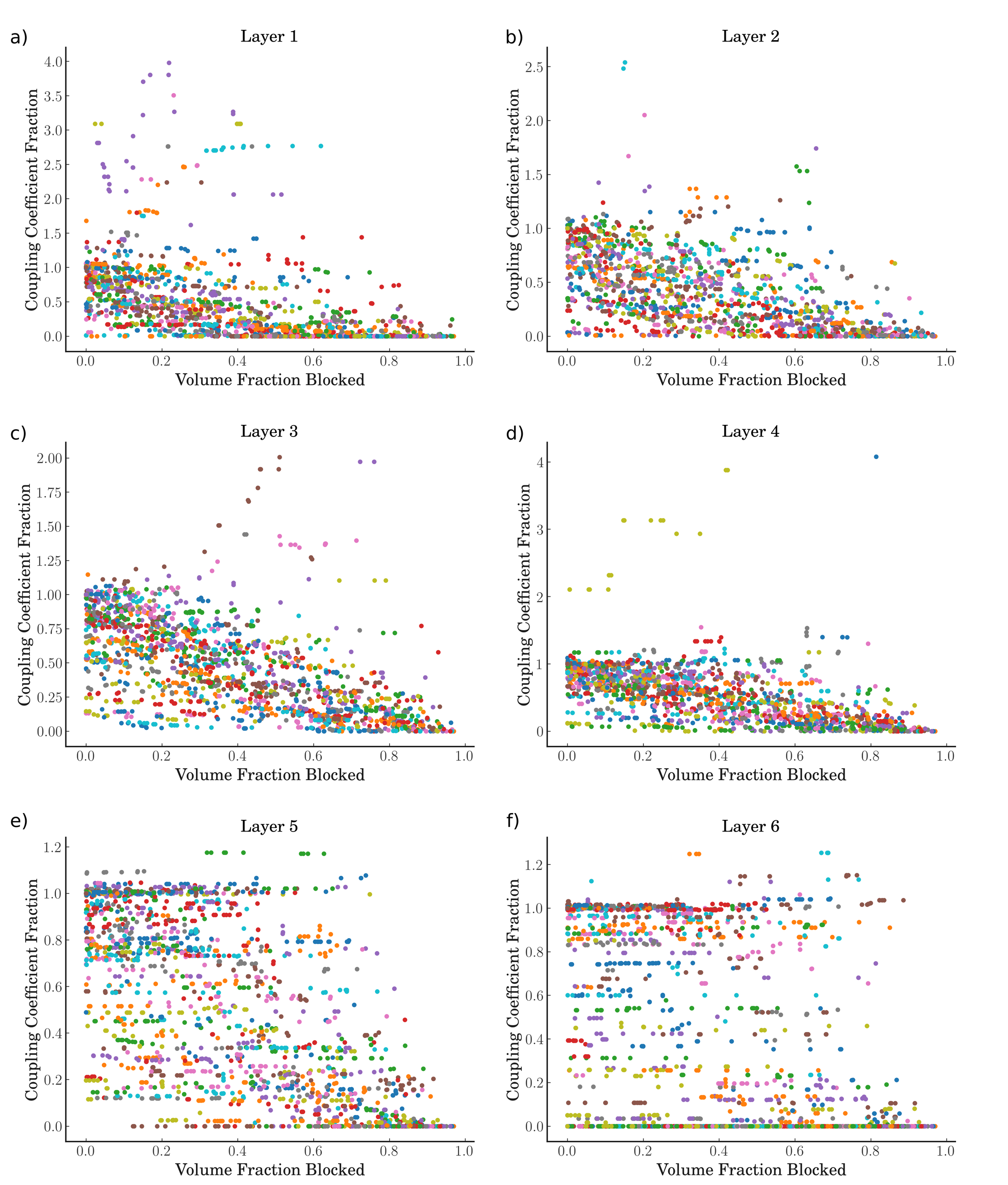

Supplement: S3 Fig — a-f) The change in coupling coefficients with respect to volume fraction blocked over the 6 layers, starting with the top layer a) and ending at the bottom layer f). (TIF) [file pcbi.1008515.s007.tif]

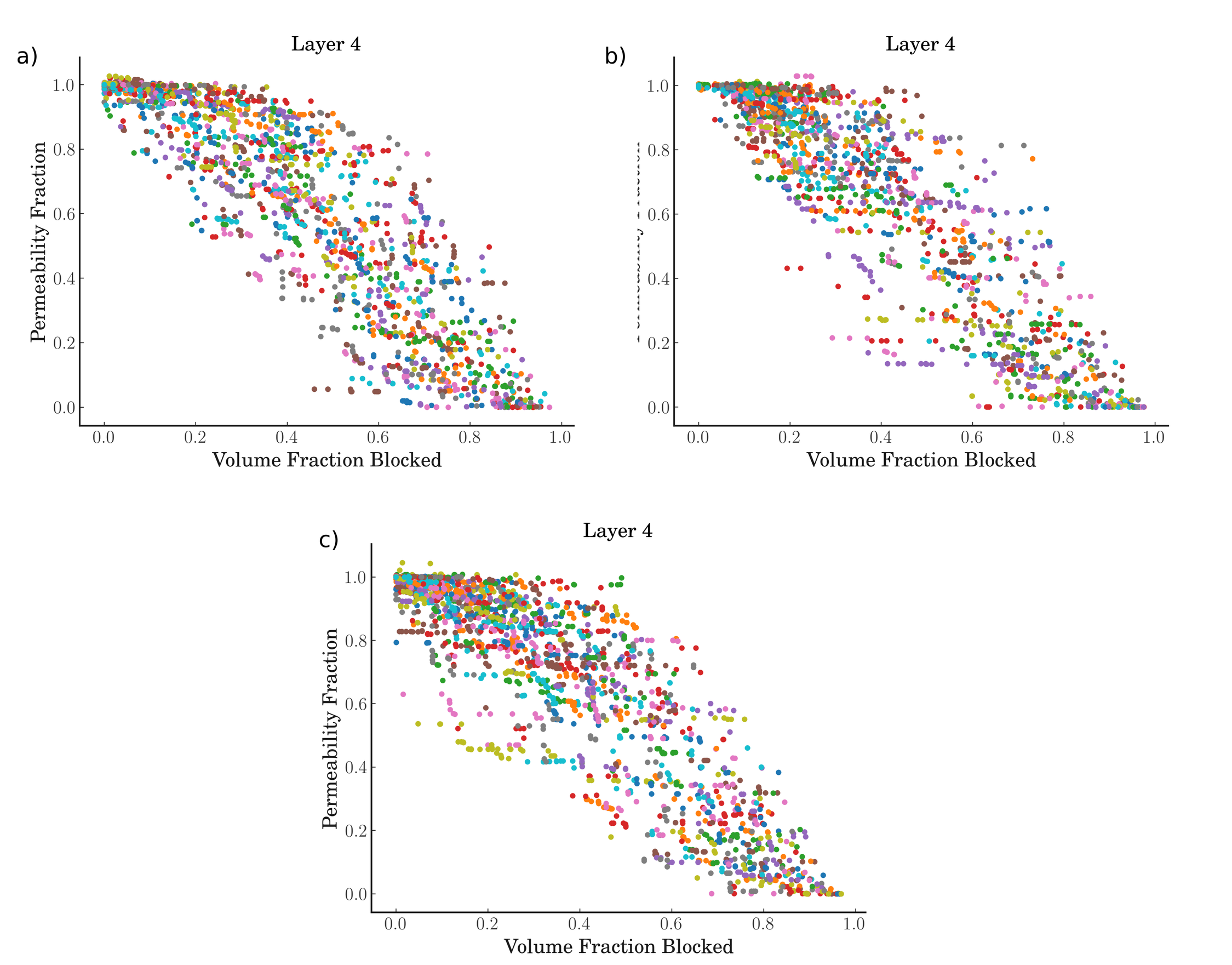

Supplement: S4 Fig — Permeability drops for 3 different sampled clot distributions over 100 voxel geometries. Results are shown for the middle layer in the voxels. (TIF) [file pcbi.1008515.s008.tif]

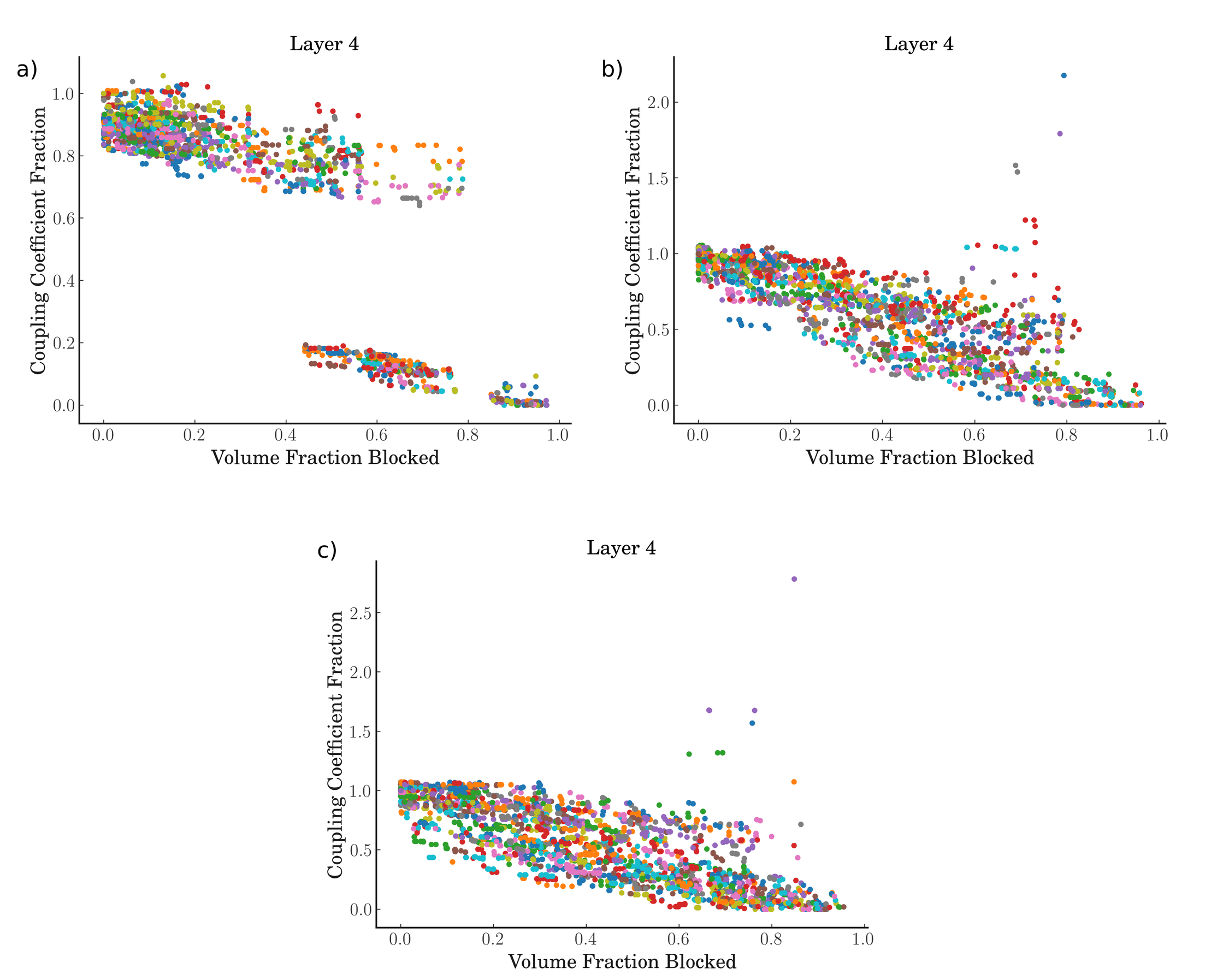

Supplement: S5 Fig — A comparison of the coupling coefficient drop against volume fraction occluded for 3 different voxel geometries simulated with 100 different clot distributions (for the ADAPT technique, hard clot) a) Voxel 1, b) Voxel 4, c) Voxel 31. (TIF) [file pcbi.1008515.s009.tif]

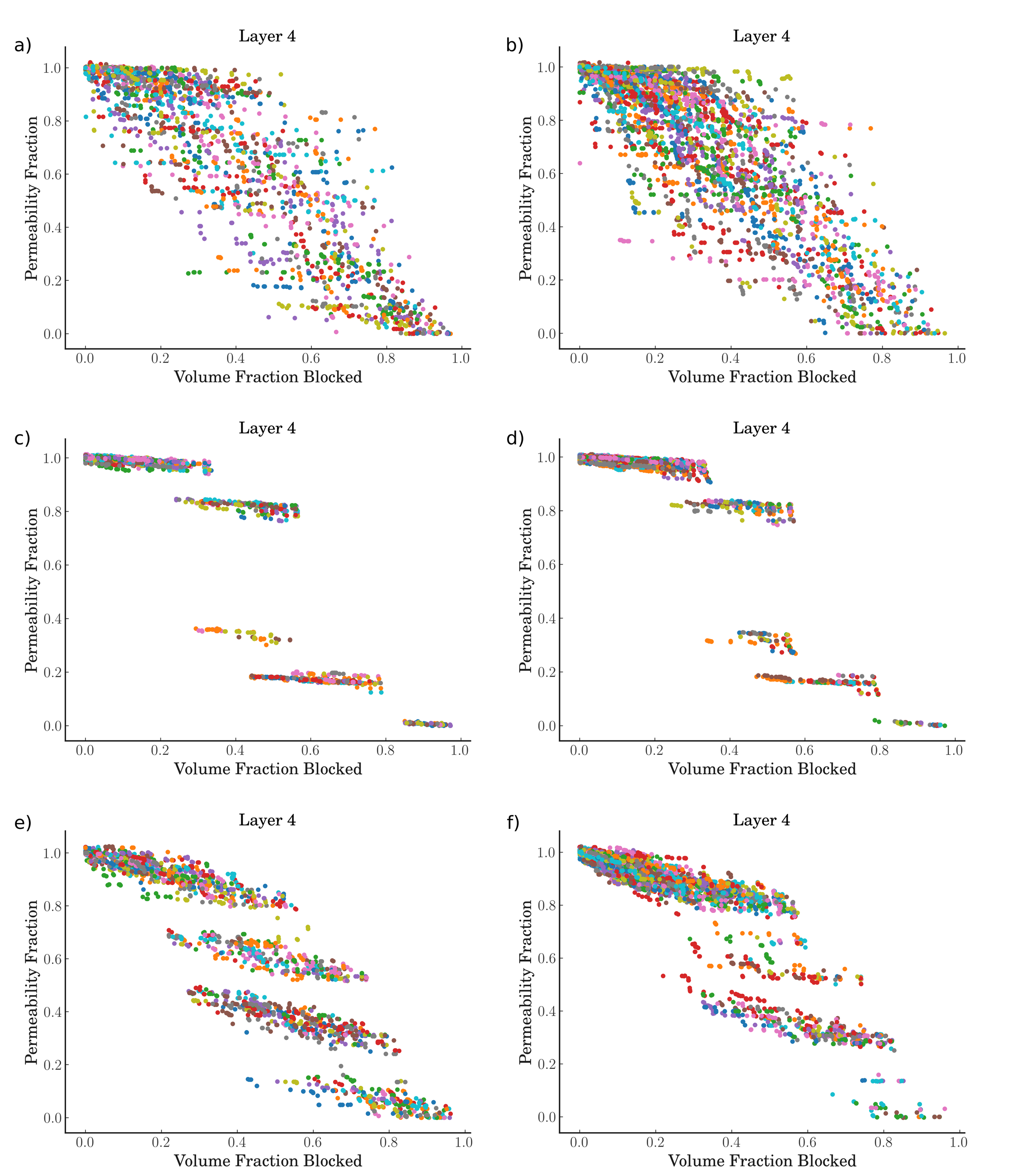

Supplement: S6 Fig — A comparison of the effect of hard and soft clots on permeability drops with respect to volume fraction occluded a) Hard clot for all 100 voxels b) Soft clot for all 100 voxels, c) Hard clot for Voxel 1 simulated with 100 clot distributions, d) Soft clot for Voxel 1 simulated with 100 clot distributions, e) Hard clot for Voxel 4 simulated with 100 clot distributions, f) Soft clot for voxel 4 simulated with 100 clot distributions. (TIF) [file pcbi.1008515.s010.tif]
